# Short-Term Culture of Human Hyalocytes Retains Their Initial Phenotype and Displays Their Contraction Abilities

**DOI:** 10.3390/cells13221837

**Published:** 2024-11-06

**Authors:** Alessandra Micera, Bijorn Omar Balzamino, Pamela Cosimi, Graziana Esposito, Guido Ripandelli, Tommaso Rossi

**Affiliations:** 1 Research and Development Laboratory for Biochemical, Molecular and Cellular Applications in Ophthalmological Science, IRCCS—Fondazione Bietti, 00184 Rome, Italy; bijorn.balzamino@fondazionebietti.it (B.O.B.); graziana.esposito@fondazionebietti.it (G.E.); guido.ripandelli@fondazionebietti.it (G.R.); 2 Surgical Retina Research Unit, IRCCS—Fondazione Bietti, 00184 Rome, Italy; pamela.cosimi@fondazionebietti.it (P.C.); tommaso.rossi@fondazionebietti.it (T.R.)

**Keywords:** hyalocytes, NGF, vitreal cells, vitreoretinal diseases, vitreous, αSMA

## Abstract

Background: Hyalocytes are the main vitreal cell types with critical functions in health and vitreoretinal diseases. Our aim was to develop cultures of human hyalocytes and verify the retention of their initial cellular features after 3 and 6 days of culturing (3 d and 6 d) by analyzing and comparing a few morphological and functional parameters. Methods: Vitreous samples (n = 22) were collected and vitreous cells and bead-enriched hyalocytes were developed and compared (3 d vs. 6 d cultures). Vitreous and conditioned media were tested for collagen, vascular endothelial growth factor (VEGF), transforming growth factor β1 (TGFβ1), nerve growth factor (NGF), matrix metalloproteinases (MMPs)/tissue inhibitors of metalloproteinases (TIMPs) and alpha-smooth muscle actin (αSMA) expression (ELISA, array/IP/WB, RT-PCR). Cells were observed at light and fluorescent microscopy (phenotypical properties) and tested for their 3D collagen gel contraction abilities. Results: An increased expression of collagens, vimentin, fibronectin, and the MMP9/TIMP1 ratio were observed in vitreous tissues. In 3 d cultures, collagens and MMP9 were upregulated while the related tissue-enzymes were deregulated. Vitreous samples also showed high levels of TGFβ1, VEGF, and NGF, and this protein signature was retained at 3 d while decreased at 6 d. The original phenotype (low αSMA) was retained at 3 d from seeding while an increased αSMA expression was observed at 6 d; NGF/trkA^NGFR^ was expressed in cultured hyalocytes and partially drives the collagen retraction. Conclusions: The vitreous print comparison between untouched and cultured hyalocytes allowed us, on one side, to select 3 d cultures and, on the other, to highlight the neuroprotective/contractile NGF in vitro hyalocytes effects. The possibility of scoring reactive hyalocytes would represent an interesting aspect of screening the vitreoretinal interface severity.

## 1. Introduction

Corroborating studies suggest that the analysis of vitreal cells collected from the vitreous during therapeutic retinal surgery (vitrectomy) can provide additional information for understanding the pathological changes occurring at the vitreoretinal interface and likewise predict local (re)activation (recidivism) [1]. The vitreous cell population includes hyalocytes, retinal pigment epithelial cells, fibroblasts, astrocytes, macrophages, myofibroblast-like cells, and eventually white blood cells, depending on the grade of inflammation [2,3]. In previous studies, we analyzed the protein profile of both the vitreous and vitreal reflux of subjects with vitreoretinal diseases undergoing vitrectomy or multiple anti-VEGF injections to reduce local inflammation and angiogenesis [4,5]. Protein profiling was prospected as reflecting the status of the underneath retina and the goodness of retinal ganglion cells (RGC) and/or photoreceptors in the presence of idiopathic and/or diabetic Epiretinal Membranes (ERMs) [6]. VEGF, NGF, and OPN were prospected as potential candidate biomarkers for the recruitment of inflammatory cells and assessment of membranogenic phases of ERMs, highlighting the participation of responsive cells (activated Müller cells, contractile myofibroblasts, and reactive microglia) [5,6]. In the last decade, attention has also shifted to hyalocytes and their ability to influence the vitreal chamber when an inflammatory process insults the retinal microenvironment [7]. Hyalocytes adhere to vitreous fibers and contribute to the entire homeostasis of the vitreal chamber [8]. Some studies reported the ability of hyalocytes to produce and bind to the extra-cellular matrix (ECM) and modulate matrix turnover throughout specific protease synthesis and release [2,3]. In line with modern thought, hyalocytes were defined as gatekeepers of vitreal chambers able to trigger and modulate the inflammatory/(para)inflammation status with the release of products of cytokines/chemokines, few oxidative-modified lipids, and products of oxidative stress [9]. In fact, hyalocytes cross-react with innate immune-specialized cells (macrophages and mast cells), myofibroblast-like cells, glia, and microglia, assuring a prompt and specific fight to biological insults, activating a proper defense and an extra-recognition of stressors (Pathogen Associated Molecular Patterns (PAMPs)/Damage Associated Molecular Pattern (DAMPs), hyaluronic acid, collagen fragments) and working for tidy regulated tissue homeostasis [6,10,11,12]. Mirroring the retinal states by the expression of specific mediators, this reservoir has recently been used for multiparametric analysis of mediators, and its usefulness as a matrix of hyalocytes for developing cell platforms for testing individual therapies (precision medicine) can be prospected soon, but this aspect would imply additional knowledge on its environment (matrix and cells) [13]. Although the process of inflammation and angiogenesis are widely documented regarding the etiopathogenesis of macular puckers [14], the contribution of hyalocytes still needs to be elucidated [15,16].

Our aim was to develop cultures of human hyalocytes and verify the retention of initial cellular features after 3 and 6 days of culturing (3 d and 6 d) by analyzing and comparing a few morphological and functional parameters. A few targets of matrix remodeling (collagens type-IX/IV, TGFβ1, VEGF, MMPs/TIMPs) and a few selected mediators of differentiation (NGF/trkA^NGFR^ and αSMA) were tested and compared.

## 2. Materials and Methods

The intramural ethical committee approved the study (IFO-Bietti, Rome, Italy; n. P.R.O. Project ver. 2.0 14 September 2024) that was performed in accordance with the ethical standards stated in the Declaration of Helsinki. Patients approved the experimental approach and signed the informed consensus for specimen collection, handling, and analysis. All chemicals, buffers, and reagents utilized in this investigation were acquired and prepared as previously published.

### 2.1. Study Population: Vitreous Samples, Hyalocytes-Enrichment, Single-Cell Separation, and Short/Long Term Culturing

All the biological samples used in this study were collected at the time of vitreoretinal surgery. Vitreous samples were harvested from 22 patients (11F/11M; 71.45 ± 7.36 years old) who underwent a pre-surgery full ophthalmic examination including anamnesis, fundus examination, and acquisition by the Spectral Domain-Optical Coherence Tomography (Spectralis SD-OCT ver.1.5.12.0; Heidelberg Engineering, Heidelberg, Germany). The inclusion criteria were idiopathic macular puckers, retinal detachment, and macular holes. The exclusion criteria included recurrent retinal detachment and anti-VEGF intravitreal injections. The characteristics of the study population and properties of vitreal samples are summarized in Table S1.

Untouched vitreous fluid was collected from the central vitreous cavity, at the beginning of the 25/27-gauge pars plana mini-invasive vitrectomy (2000 starting cuts). The organoleptic features of the sampled vitreous can be defined in terms of the jelly-to-liquid appearance, volume (250–800 µL), and total protein content (150 ng/µL). The vitreous was left at room temperature for 15 min to allow sedimentation and supernatants were gently removed by centrifugation (1500 rpm for 3 min). Pellets including cells and matrix were gently resuspended in a 15 mL conical tube containing Iscove’s modified Dulbecco’s medium (IMDM; Waltham, MA, USA) supplemented with 10% fetal bovine serum (FBS), 1% glutamine, and 100 units/mL mix penicillin/streptomycin [15]. To increase the rate of hyalocyte isolation and maximize the beads-separation, the vitreous was further incubated at 37 °C under gentle shaking in the presence of collagenase (1 mg/mL; Sigma, Milan, Italy) followed by dispase II for an additional 30 min. After enzymatic digestion, the vitreous bodies were centrifuged at 300× *g* for 5 min and pellets were used for bead-driven cell separation (antiCD45 and antiCD11a cocktail), according to a standard procedure [15]. Cell-culture biodegradable beads were conjugated with a mix of CD45 and CD11a antibodies 1:10 diluted in PBS containing 0.05% Tween (PBST) and left at room temperature for antibody–bead complex formation according to a standard procedure [15]. For separation, cells were placed in a medium and incubated at 37 °C in the presence of 5% CO_2_ with partial medium refreshing every day (1:1; vitreous medium). In preliminary tests, different media and different enzymatic procedures were tested according to the literature. For these studies, untouched or enriched/selected cells were seeded in collagen-coated 24-well plates (1000 cells/250 μL) in the presence of 1:1 vitreous medium, and after, 3 d cells were sampled or shifted in IMDM (1% FBS supplemented Iscove’s Modified Dulbecco’s Medium containing 1% antibiotic mix) until 6 d.

**Figure 1:**
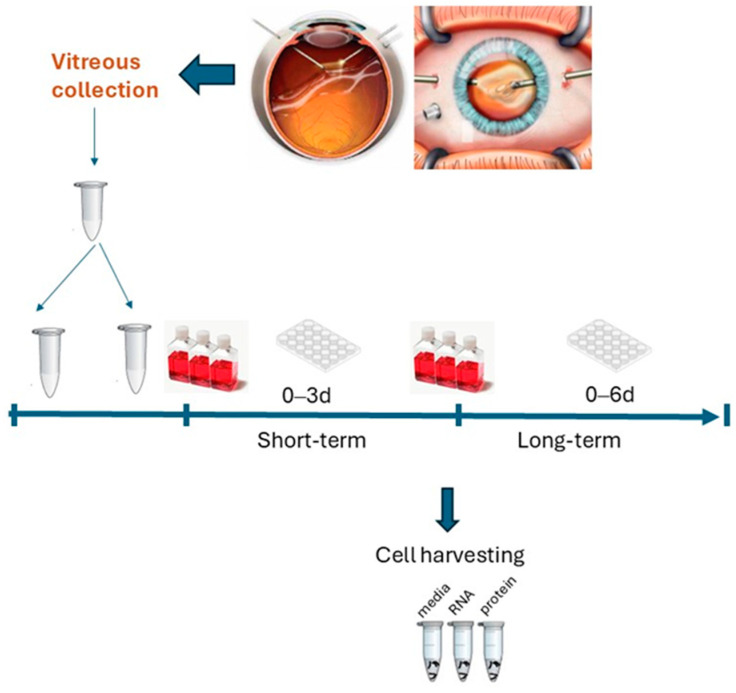
Flow chart of the study. Hyalocytes were extracted from vitreous samples collected at the time of vitreoretinal surgery. Enrichment and bead-based selection were carried out to favor the growth of hyalocytes in a media resembling those of a vitreal chamber (1:1 vitreous-DMEM) shifted after 3 d into complete IMDM. Cultures were directly used for light microscopy or harvested for RNA and protein analysis. Conditioned media from 6 d, 3 d, and 0 d were compared. Adherent cells were quickly fixed in 2% buffered PFA while single cells were directly extracted in lysis solution and devoted to RNA/protein extraction. Conventional detachment with trypsin-EDTA was not used to avoid cell loss at pre-washing and centrifugation.

A scheme summarizing the entire experiment is shown in Figure 1.

### 2.2. Proliferation, Survival, and Mechanical Assessments

To evaluate cell viability, monolayers were seeded in 96-well culture plates (500 cells/200 μL) and exposed to viability tests after 1 and 3 h from harvesting (0 d, 3 d, 6 d). Adherent cells were washed in HBSS and stained with cresyl violet, a dye staining the DNA. The quantification was carried out by measuring the absorbance at 562 nm, after dye solubilization with 10% acetic acid. As an additional check, the monolayers were exposed to trypan blue and observed under light microscopy. The presence of blue-stained death cells was assessed by the Real Time-Glo™ MT Cell Viability Assay (G9711; Promega, Madison, WI, USA). In some experiments, a Trypan blue solution was used, and cells were counted in a Burker chamber, according to a routine procedure.

For rating apoptosis, two microscopical techniques were used for discrimination between apoptosis and necrosis. Briefly, adherent cells were stained with DAPI, to identify chromatin condensation and clumping, a characteristic feature of apoptotic cells, by a blinded observer. The percentage of apoptosis was calculated as follows: [(apoptotic cells’ number/total cells’ number) × 100]. Sister cells were stained with Hoechst 33258 (1 µg/mL/15 min, 37 °C; Invitrogen-Molecular Probes) and counterstained with PI. Both techniques were used in previous cell culturing studies [17].

To evaluate cell contractility, vitreal cells (1 × 10^3^ cells/200 μL) were resuspended in 0.5% FBS IMDM either alone or supplemented with 50 ng/mL NGF as a stimulating factor and seeded in 24 well plates containing round-coverslip and a 1% collagen solution containing trypan blue [18]. The unit of contraction was estimated every day till 6 d. At 3 d and 6 d, the gels were fixed in buffered PFA and imaged at 20× under an inverted light microscope (Nikon Imaging Japan Inc., Nishioi, Shinagawaku, Tokyo, Japan).

### 2.3. Immunofluorescence and Confocal Analysis

To confirm that these 3 d cultures were mainly hyalocytes, the immunofluorescent staining was performed using specific antibodies recognizing CD64 [19,20]. Alternative checks were carried out with specific antibodies recognizing CD67. Briefly, untouched and enriched sorted cells were seeded as a whole-mounted drop on a pretreated glass slide (BDH, Milan, Italy), postfixed with cytofix (Cyt solution, Hologic, Inc., Marlborough, MA, USA) and air-dried. Postfixed cells were rehydrated and processed for basal staining or probed for immunofluorescent analysis. Round coverslips (24 well-plate) were analyzed after appropriate time points and attached cells were fixed in 4% buffered paraformaldehyde (PFA-PBS; 1xPBS: 10 mM Na_2_HPO_4_, 1.8 mM KH_2_PO_4_, 137 mM NaCl; pH 7.4) for 10 min at room temperature. Cells were then washed with 1XPBS for 5 min, incubated with blocking solution (4% BSA in PBS 1X) at room temperature for 30 min, and incubated with the following fluorochrome-conjugated antibodies: CD45 fluorochrome-conjugated antibody (2D1, 1:100 in 2% BSA), CD11a (1:40 in 2% BSA), and CD64 (1:100 in 2% BSA), all from Thermo Fisher Scientific (Waltham, MA, USA) or Abcam (Cambridge, UK) or Santa Cruz (Santa Cruz, CA, USA). As exclusion markers, we use the following: anti-ionized calcium-binding adapter molecule 1 antibody (Iba1; mouse; 1:500; Santa Cruz) and CD68 and anti-Glial Fibrillary Acidic Protein antibody (GFAP; mouse; 1:500; Cell Signaling, Denver, MA, USA). Washed coverslips were mounted with a mixture containing nuclear stainer (DAPI or PI) diluted 1:10,000 in 2% BSA mounted in an anti-fading solution.

To characterize 3 d cultured cells in terms of matrix and growth factor expression, conditioned media and harvested hyalocytes were processed for protein and RNA extraction as shown below in separate paragraphs.

For validating the NGF response, coverslips were equilibrated (10 mM Phosphate Buffer (PB) and 137 mM NaCl; pH 7.5), blocked, and permeabilized (0.1% BSA and 0.3% Triton X100 in PBS) before antibodies’ incubation: anti-human CD45/CD11a/CD64, anti-human α-Smooth Muscle Antibody (αSMA; mouse; 1:500; Sigma-Aldrich, St. Louis, MO, USA), anti-ionized calcium-binding adapter molecule 1 antibody (Iba1; mouse; 1:500; Santa Cruz), and anti-Glial Fibrillary Acidic Protein antibody (GFAP; mouse; 1:500; Cell Signaling, Denver, MA, USA). Specific bindings were detected using secondary AlexaFluor-555- or AlexaFluor-488-coupled anti-rabbit (for CD45) or anti-mouse (αSMA, Iba1, GFAP) species-specific F(ab)_2_ antibodies diluted (1:500) in 0.05% Tween20-PBS and incubated for 45 min on a benchtop (Immunological Sciences, Rome, Italy). Antibodies are summarized in Table S2A. After nuclear counterstaining (blue/DAPI; Invitrogen Molecular Probes, Waltham, MA, USA), slides were mounted with anti-fading supplemented Vectashield (Vector Laboratories, Inc., Burlingame, CA, USA) and examined under an epifluorescent direct microscope (Ni-Eclipse) equipped with a UV lamp, digital camera (Axiocam), and NIS-Elements software F 4.00.00 for digital assets (8-tiff format; Nikon, Tokyo, Japan). Acquisitions were carried out at ×20 and ×40 objectives, both in single and merged image settings.

### 2.4. Transcription Analysis: cDNA Synthesis and Real-Time PCR

The total RNA was obtained from vitreal fluid untouched, untouched/enriched, and cultured cells by using TRIzol procedure (Thermo Scientific Pierce, Waltham, MA, USA). The total RNA was dissolved in 11 µL RNAse free water (depc-treated and autoclaved MilliQ water, Millipore, Waltham, MA, USA) and spectrophotometrically quantified (Nanodrop, Thermo-fisher Scientific). Retro-transcription (50 ng total RNA) was carried out by using the Excel-RT Reverse Transcription polymerase (SMOBIO Technology, Inc., Hsinchu City, Taiwan). The cDNA synthesis (3 µL/target and 1 µL/referring gene) was performed in a LifePro Thermal Cycler (EuroClone, Milan, Italy) while the specific amplifications were carried out in a Biorad CFX96 Real-Time PCR System (BioRad., Hercules, CA, USA) by using the Hydra SYBR green Hot start PCR Master Mix (39-cycle amplification program; Biocell, Rome, Italy). Negative and positive controls (pools) were used for validating PCR amplifications, and a pool of controls was used for the relative gene expression analysis. The specificity of amplifications was confirmed with the elaboration of the melting curves at the end of each amplification and the evidence of a single amplicon (band). Cq values from normalized samples were used only if coming from specific amplifications lower than 35 cycles of replication. Changes in gene expression were provided as a log2 expression ratio with respect to the referring group, considering four different housekeeping genes. Primer pairs were synthesized by Eurofins MWG Genomics (https://eurofinsgenomics.eu/, accessed on 26 February 2024) Supplementary Table S2B.

### 2.5. Biochemical Analysis: Immunoprecipitation and Western Blot Analysis (IP-WB) and ELISA Assay

The conditioned media were collected and subjected to ELISA or a protein chip array to quantify the collagen, metalloproteinases, and growth factor (NGF, VEGF, and TGFβ1) in 3 d- and 6 d-cultured cells. The detection of native proteins was assessed in 1:2 diluted VH and conditioned media aliquots (see [5,6]). The total proteins were initially quantified by using the nanodrop spectrophotometer. For ELISA analysis, the protocols were as reported by the manufacturers (NGF (DY256-05) detection; R&D Systems, McKinley Place, Minneapolis, MN, USA). For immunoprecipitation and immunoblotting, Magnetic Beads (Protein A Magnetic Beads; (Protein A Magnetic Beads; Thermo Scientific Pierce, Waltham, MA, USA) were used with antibodies listed in Table S1A [21]. Specific antibody–bead–protein complexes were eluted in 2× Loading-Buffer (Invitrogen) supplemented with β-ME, boiled (98 °C/5 min), and electrophoresed in 4–20% SDS-PAGE minigels (miniprotean; Biorad, Hercules, CA, USA). After separation, gels were stained according to a standard protocol (SYPRO Ruby gel stain; Thermo Fisher, Waltham, MA, USA) and acquired in a B-BOX Blue Light LED epi-Illuminator (Smobio, Hsinchu City, Taiwan). Gels were stained for specific targets and reprobed for a referring one (GAPDH). Band analysis was performed by using ImageJ v1 (Rasband, W.S., ImageJ, U. S. National Institutes of Health, Bethesda, MD, USA, https://imagej.net/ij/, 1997–2018 accessed on 12 September 2024).

### 2.6. Statistical Analysis

Descriptive statistics included mean ± SEM or mean ± SD for continuous measurements (Prism10.0; GraphPad Software Inc., San Diego, CA, USA). The normal distribution of data was tested by using the Kolmogorov–Smirnov and Shapiro–Wilk’s tests, confirming the absence of a normal distribution of laboratory parameters. Therefore, Mann–Whitney’s U test was applied for the following analysis. The REST-ANOVA coupled analysis was carried out to identify significant changes in the transcripts’ expression [22]. Correlations were assessed between subgroups using the Pearson rho correlation test, after a normalization check. The significant levels were *p* < 0.05 (*), *p* < 0.01 (**), and *p* < 0.001 (***).

## 3. Results

Herein, 22 vitreous samples were used to isolate, culture, and characterize hyalocytes (500 cell/mm^2^; 20%) and to assess their possibility of still expressing some biomolecular targets upon 3 d and 6 d of culturing.

### 3.1. Organoleptic Features of Vitreal Fluids and Cell Density

The normal vitreous appeared as a gel populated by few cell types mainly located in the cortex (hyalocytes, astrocytes, and glial cells), while upon vitreoretinal disease onset, the organoleptic properties changed to opacification, liquefaction, and shrinkage. The organoleptic characteristics of these samples have been registered and are summarized in Supplementary Table S1. The variability between samples in terms of cell number, subtype, and morphology (microglia, macrophages, and even mast cells and other inflammatory cells) is strictly dependent on the disease origin. Depending on the grade of inflammation, vitreal samples displayed different levels of jelly to watery appearance. As shown in Supplementary Table S1, a total of 8/22 displayed a jelly appearance as 8/22 showed different levels of liquefying and finally 6/22 were completely fluid. The average volume was 600 ± 200 µL, ranging from 250 to 800 µL, while the protein amount was increased in liquified samples (total protein average 150 ng/µL, ranging from 100 to 200 ng/µL). Table S1 also summarizes the cellular density averaged between 200 and 600 cells/mL that represents the pellet, with a cell composition depending on the type of inflammation, in line with previous studies [15]. For cell dissociation and enrichment, two enzymes were tested for dispase II and trypsin solution [23]. The advantages of dispase II were clear in 15/22 cultures, showing a <5% dead cell detection.

### 3.2. Hyalocyte-Enriched Cultures: Untouched and Bead-Driven Cell Separation for 3 D and 6 D Cultures

Cells were seeded (1000 cells/well) in 96 well tissue-culture plates and collected at 3 d and 6 d from initial plating (baseline; 0 d). Finally, cells were embedded in a gel matrix and their ability to contract the matrix was tested in the presence or absence of NGF as a stimulating factor.

**Figure 2:**
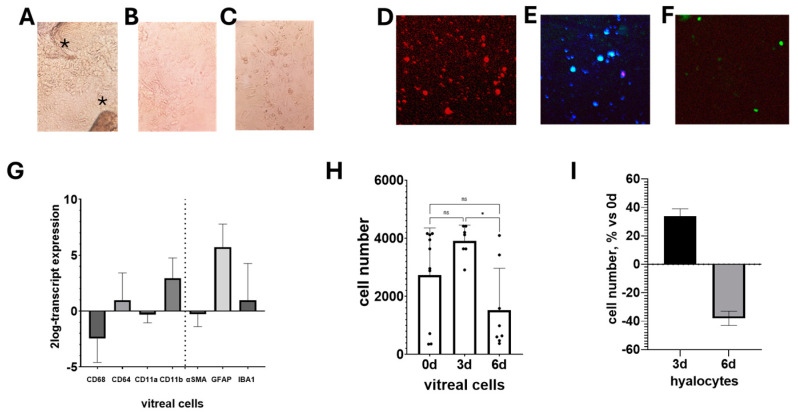
Human hyalocytes in 3 d and 6 d cultures. Phase contrast microscopy of monolayers acquired from vitreous cells (**A**–**C**) and hyalocytes (**D**–**F**) cultured and analyzed after 3 d and 6 d from seeding (0 d). In the light microscope acquisitions, no stains nor dyes were used for evaluation (**A**–**C**). Note the different cell densities depending on the characteristics of the specimens (**A**–**F**). (×200, original magnification) Untouched cells displayed different markers of macrophage lineage (**G**) as detected by PCR analysis. A selected transcript analysis of vitreous cells displays a typical pattern of differentiation (**G**). The cell survival profile of enriched vitreous cells (**H**) showed an increased cell number at 3 d and a significant reduction at 6 d (**I**) with respect to 0 d. The decrease between 6 d and 3 d was significant (*p* < 0.05; ns not significant, ANOVA Tukey–Kramer post hoc). Bead-selected hyalocytes showed an increase at 3 d and a decrease at 6 d from culturing, with respect to 0 d (**C**). * *p* < 0.01.

Light microscopy displayed the fact that vitreal fluids, collected at the time of surgery, contain a mix of several cell types (Figure 2A) and these cells enrich after 1 h incubation at 37 °C before plating (Figure 2B). The total amount of cells in the vitreous was 3432.50 ± 361.55 cells, ranging from 3633 to 5997 cells. The higher cell density was observed after enrichment and after 3 d of culturing (4733.30 ± 838.07; 1468 and 2170 cells/mm^2^; Figure 2A,B) with respect to 6 d (4588.30 ± 605.75; 34 and cells/mm^2^; Figure 2C,D). Different cell types populate these primary cultures displaying typical round to spindled shapes (Figure 2C). Cells with a myofibroblast appearance were also visible in a low-to-high density plating (Figure 2D). An initial staining displays the presence of some cells expressing CD45/CD11a markers (Figure 2E,F). In line with previous studies, CD11a and CD45 positive hyalocytes were observed in untouched vitreal cells (Figure 2E,F). The use of bead separation demonstrated the feasibility of these cells that prefer a more selected or cell-enriched environment (Figure 2). Cellular density averaged between 200 and 600 cells/mL (pellet), with a cell composition depending on the grade of inflammation and disease. Indeed, 3 d cultures showed a 73% percentage of viable cells (SD 29%; range between 17% to 95%) while 6 d cultures showed a reduced 47% percentage of viable cells (SD 59%; range between 12% to 84%). The MTT assay showed that up to 1250 μg/mL did not raise effects on cell viability, although a slight reduction in cell proliferation was observed in 3 d and 6 d cultures. The number of apoptotic cells was restricted to 20% of total cells (Figure 2H,I).

**Figure 3:**
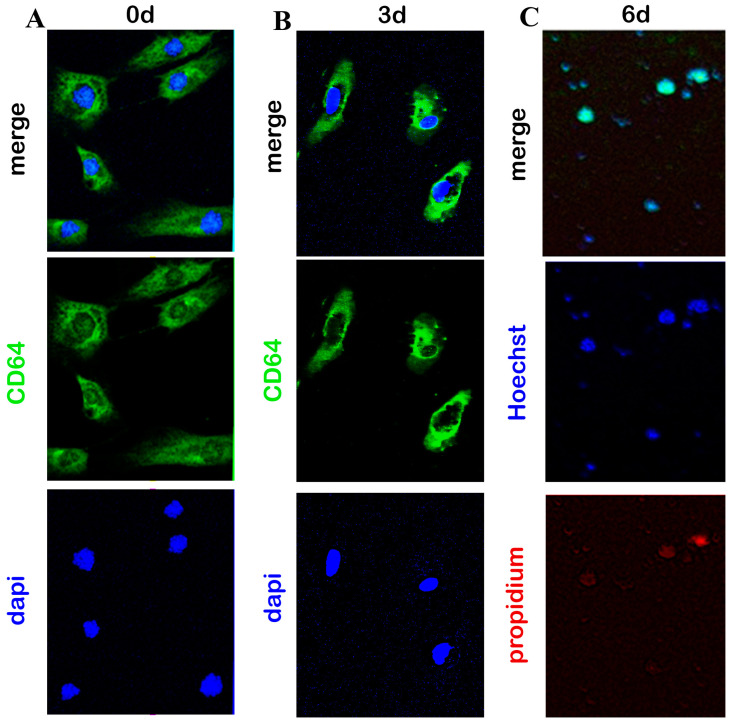
Immunophenotypical characterization. Epifluorescent images of hyalocytes double-stained with CD64/dapi (**A**) or triple-stained with CD64/Hoechst/propidium (**B**). Cells were seeded (1000 cells/well) in 24 well tissue-culture plates and collected at 3 d and 6 d from initial plating (baseline; 0 d). Adherent cells were postfixed and immunostained. Representative images of bead-separated hyalocyte (CD45/CD11a/CD64) cells cultured on round coverslips and imaged at 3 d (**B**) and 6 d (**C**) from initial plating (**A**). Overlay images (upper) display immunoreactivity at 0 d (**A**), 3 d (**B**), and 6 d (**C**); middle layers show CD64 (cy2/green) immunoreactivity changes and lower panels show nuclear intercalants (Hoechst/dapi/blue). (Original magnification ×400).

More enriched hyalocyte cultures than untouched ones were more suitable to be useful for bead-driven separation. The use of leukocyte-associated antigens CD45 and CD11a was able to select relatively pure hyalocytes for plating [19,20]. Since other studies reported that cultured hyalocytes also express CD64, a constitutive target of monocytes and macrophages, and do not react to CD68, an antigen expressed by tissue macrophages, our cultures were probed with anti-CD64 antibodies [19,20]. As shown in Figure 3, bead-selected cultures displayed CD64 at 3 d (A) and 6 d (B) post-culturing. Of interest, the morphology of these cells changed from spindle (3 d) to round (6 d), indicating some morphological changes occurring at plating. 

### 3.3. Enriched Hyalocytes Display Changes in ECM Expression

**Figure 4:**
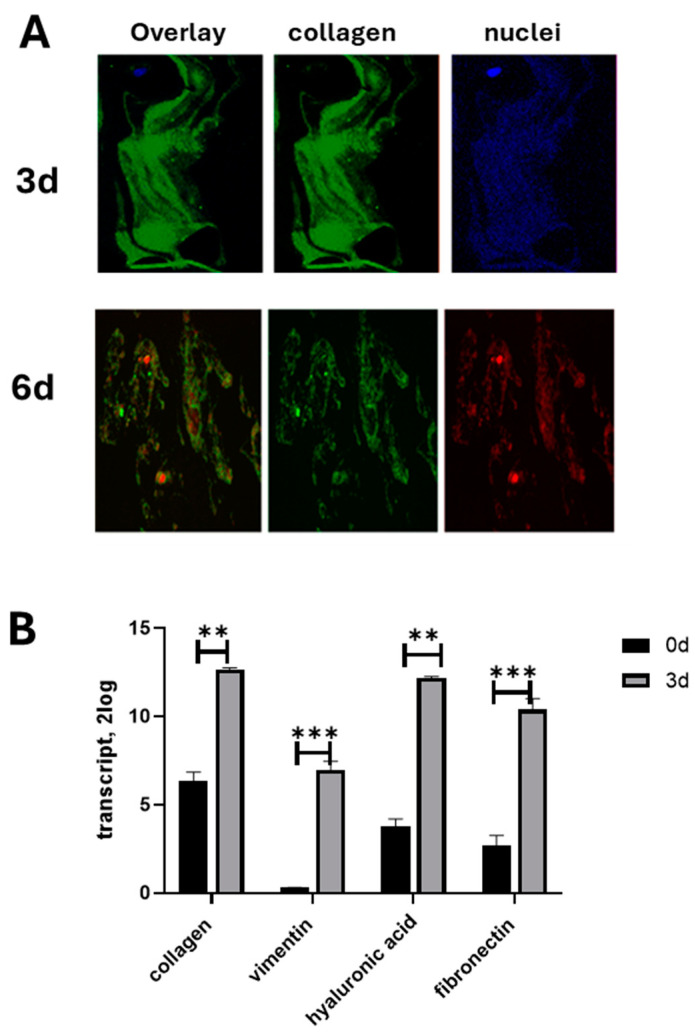
Hyalocyte cultures partially retain their original phenotype. Representative epifluorescent images of vitreous cells as double-stained with fluorescent collagen type IX marker and nuclear intercalant (DAPI/3d or PI/6d) at 3 d and 6 d time-culturing (**A**). Cells were seeded (1000 cells/well) in 24 well tissue-culture plates and postfixed at 3 d and 6 d from initial plating (baseline; 0 d). Note the decreased immunoreactivity of collagen matrix after 6 d of culturing. Single acquisitions for collagen type IX are shown in the middle panels (cy2/green). (Original magnification ×200). Transcript analysis showed a significant upregulation at 3 d from plating (**B**). Note the transcription expression of collagen type-IV, vimentin, hyaluronic acid, and fibronectin transcripts’ expression as assessed by REST-Anova analysis (**: *p* < 0.001; ***: *p* < 0.0001).

Hyalocytes populating the vitreous chamber are the main cell types producing collagen and other matrix components, including matrix enzymes (MMPs) and related inhibitors (TIMPs), that provide the characteristic jelly feature to the vitreous [3,24]. As shown in Figure 4A,B, a significant expression of procollagen was observed at plating while a reduction in the immunostaining was observed at 6 d (Figure 4A).

Matrix protein transcripts (collagen, fibronectin, and vimentin) were significantly upregulated at 3 d (Figure 4B) while significantly reduced at 6 d of culturing.

### 3.4. Hyalocyte-Enriched Cultures Express Growth Factors Retaining Their Initial Pathological Phenotype in 3-D Cultures and Express the NGF Pathway

**Figure 5:**
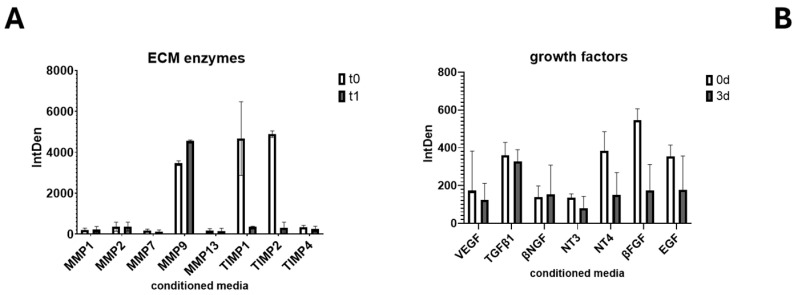
Tissue enzymes and growth factors are deregulated in 3 d cultures. Analysis of ECM at plating showed the presence of metalloproteinases’ enzymes (MMPs) and related tissue inhibitors (TIMPs) (**A**). An increased expression was observed for MMP9 at 3 d of culturing, while a significant decrease was monitored for TIMP1/2 (**A**). Growth factors analysis showed no significant changes in NGF expression while VEGF, TGFβ1, and the other neurotrophins were deregulated (**B**). ANOVA Tukey–Kramer post hoc analysis.

Since growth factors play a crucial role in hyalocyte growth, survival, and mediator release, a protein array was devoted to screening the expression of a few selected mediators of remodeling. As shown in Figure 5, conditioned media analysis showed a significant expression of MMP9 at 3 d in association with an opposite expression of tissue inhibitors TIMP1 and TIMP2 (Figure 5B). High levels of growth factors (VEGF, TGFβ1, NGF) were assayed at baseline. Particularly, a reduced expression of VEGF and TGFβ1 as well as βFGF and EGF was monitored after 3 d of culturing. Of interest, NGF did not show changes during plating (0–3 d), a dissimilar effect considering the decreased expression of NT3 and NT4.

**Figure 6:**
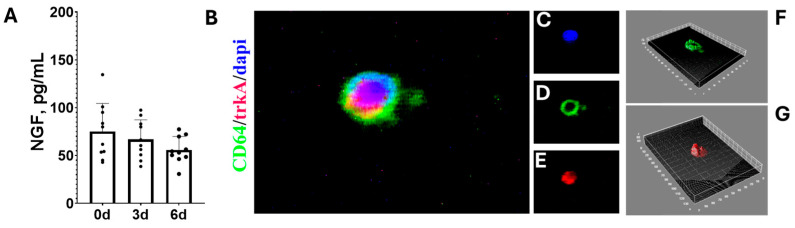
NGF and trkA^NGFR^ expression as a function of culturing. Cells were seeded (1000 cells/well) in 24 well tissue-culture plates and harvested at 3 d and 6 d from initial plating (baseline; 0 d) for NGF quantification or they were immunostained for trkA^NGFR^ examination. (**A**) NGF protein expression as a function of culturing (0 d, 3 d, and 6 d; ANOVA Tukey–Kramer post hoc). Representative epifluorescent images of 3 d hyalocytes (CD45 and CD11a pos selection) cultured for 3 d and identified by nuclear counterstained with dapi (blue; (**B**)) and double-immunostained for CD64 (green) and trkA^NGFR^ (red). As shown in overlay (**B**), CD64 was mainly localized at the membrane level (green; (**D**)) and equally distributed, while the trkA^NGFR^ receptor appears in membrane zones (red; (**E**)), nuclei were stained with dapi (**C**). This distribution cluster of trkA^NGFR^ is more visible in the interactive 3D surface plot compared to the CD64 uniform distribution ((**F**) vs. (**G**); ImageJ). The nuclear localization by DNA intercalant (dapi/blue) is suggestive of signal specificity. Original magnification ×400 for Merge and single acquisitions.

The expression of NGF was accurately investigated. As shown in Figure 6A, the content of NGF did not change between time points, but a trend to a decrease was observed. Three-day-cultured hyalocytes expressed trkA^NGFR^ as shown by immunofluorescent analysis (Figure 6B). The IntDen analysis showed a trkA^NGFR^ distribution in clusters over the hyalocyte membrane (Figure 6C,E), as observed by comparing the distribution of CD64 (Figure 6C,D). The interactive 3D surface plot highlights this redistribution of trkA^NGFR^, indicative of the presence of 3 d hyalocytes reactive to NGF (Figure 6F,G).

### 3.5. Cultured Hyalocytes Retain the Ability to Contract the Collagen Matrix

**Figure 7:**
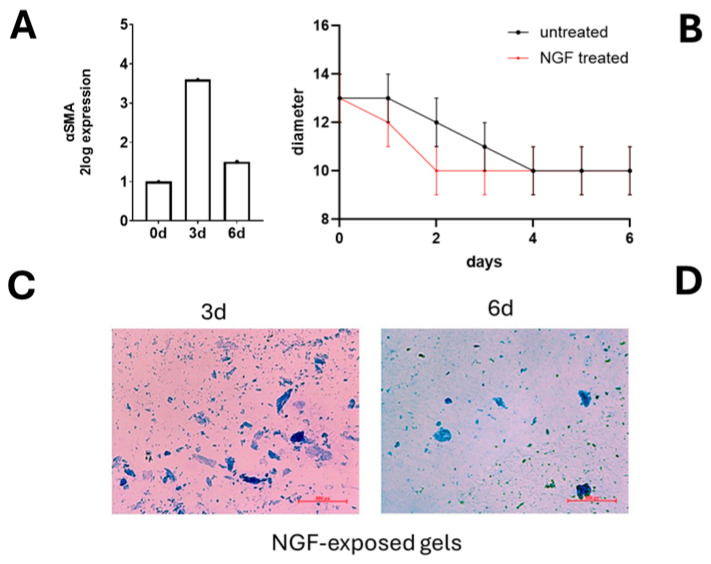
Hyalocytes express αSMA phenotype and contract a gel matrix upon NGF stimulation. Cells were embedded in a gel matrix (see MM for details) and their ability to contract the matrix was tested in the presence or absence of NGF as a stimulating factor. (**A**) Hyalocyte RNAs from 3 d- and 6 d cultures were probed for αSMA. A significant transcription of αSMA was observed at 3 d and quickly reduced at 6 d from plating (0 d). (**B**–**D**) Vitreous cells were drawn off and embedded in a 3D gel contraction pre-stained model. Untreated (black-line) and NGF-supplemented (red-line) vitreous cells were monitored daily for 6 d. Diameters were daily measured, and the results are plotted in (**B**). Gel retraction occurred by 3 d culturing and was absent at 6 d of culturing. As expected, the range of contraction was strictly related to the αSMA transcription. Representative images from 24 well-plates have 3 d cell-driven retracted gels (**B**).

Since a common characteristic of vitreoretinal disorders is retinal retraction, hyalocytes were tested for their potential ability to in vitro contract an ECM scaffold (retained ability to contract a collagen matrix). As shown in Figure 7A, cultured hyalocytes harvested at 3 d showed an increased expression of transcripts specific for αSMA, a classic myofibroblast marker, which decreased at 6 d, reaching quite the initial expression. A 3D collagen gel contraction test was thereafter performed in the presence or absence of supplemented NGF. As shown in Figure 7B, hyalocytes were able to retract the collagen matrix either alone (back line) or in the presence of exogenous NGF (red line). The addition of NGF was used as an alternative to TGFβ1 as a positive control. This hyalocyte reactivity to NGF exposure in a 3D-gel contraction system is shown in Figure 7B. Representative gel-embedded hyalocytes at 3 d and 6 d are shown in Figure 7C,D.

## 4. Discussion

Herein, we extended previous studies from Nuzzi et al. showing the possibility of developing 3 d cultures of enriched hyalocytes with the ability to contract a 3D gel matrix (αSMA/3D contraction). Our data confirmed the retention of initial pathological features of hyalocytes after 3 d of culturing, a mandatory aspect in the case of the setup of a cell model for diagnostic purposes. The validations of these cell cultures were carried out at both biochemical and molecular levels by using a few specific clusters of differentiation (CD45, CD11a, CD64) and analyzing some matrix (collagen, MMPs/TIMPs) and a few selected pro-inflammatory/fibrogenic/neurotrophic (VEGF, TGFβ1, NGF/trkA^NGFR^) targets. The novelty of our study is that these 3 d cultures preserved the same characteristics of commencing fluid and appear suitable for studying the physiopathology of the vitreous [15,16].

The normal vitreous appears as a jelly-like fluid populated by a few cell types mainly located in the cortex (hyalocytes, astrocytes, and glial cells), while it changes upon vitreoretinal diseases in terms of organoleptic properties (opacification, liquefaction, and shrinkage) and cell number, subtype, and morphology (microglia, macrophages, and even mast cells and other inflammatory cells), as reported from the observation of vitreous samples from retinal detachment and epiretinal membrane formation [25,26]. Pathological vitreous displays a specific protein signature due to the accumulation of mediators depending on the pathological states (degenerative, diabetic, autoimmune), and cadaveric vitreous cannot be considered “healthy-like fluid” as it also displays a divergent protein signature [26,27]. In this immune-privileged cavity (vitreous cavity-associated immune deviation), hyalocytes play the role of antigen-presenting cells, drive the maintenance of vitreous structure/composition as an avascular and transparent tissue, and are probably involved in vitreoretinal impairments (epiretinal membrane formation) [3,25,28]. Functionally, hyalocytes can be categorized for i. synthesis of extracellular matrix; ii. regulation of the vitreous cavity immunology and modulation of inflammation, and iii. retraction of the vitreoretinal interface [25,28]. Working as gatekeepers at the vitreous interface, hyalocytes drive some of the physiological functions of the vitreous body and participate in the pathological states [7,28]. Under physiological conditions, the hyalocytes are the only cells within the vitreous body, while during inflammatory states, hyalocytes cooperate with other cell types in a tidy crosstalk [3,7]. Although hyalocyte origin, phenotype, and functional characteristics are known, additional knowledge about these cells could be the key to a better understanding of the pathophysiology of the vitreous body [29]. Inflammatory stimuli trigger hyalocyte proliferation, and this proliferative activity was displayed in vitro in response to inflammatory molecules and leads to the secretion of VEGF and other activators [28]. Understanding the properties of hyalocytes might be important to comprehend the biology inside the vitreous cavity and to develop novel treatments for vitreoretinal diseases [15,28].

First, we developed, as described, a minimal protocol to develop cultures of primary hyalocytes from human pathological vitreous collected at the time of vitrectomy. Hannover (1840) was the first to describe vitreous cells as hyalocytes and Nuzzi et al. (2000) developed and characterized primary cultures of human hyalocytes [15,30]. The difficulty in developing primary cultured hyalocytes is clear, especially if the goal is retaining an initial pathological phenotype [2,15]. Since hyalocytes appear to be intimately associated with cortical vitreous fibers, we utilized the enzymatic treatment to reduce the strength association of these cells to the collagen fibers [15,16]. The use of collagenase followed by dispase II was practical to provide single cells for bead separation. The choice of CD markers in the literature was based on hyalocytes belonging to the monocyte/macrophage lineage as they express CD45 (leukocyte common antigen), CD11a (lymphocyte function-associated antigen), CD64 (Fc-gamma receptor 1), and CD63 (pan-macrophage receptor); whereas, they do not react with antibodies against CD68 (antigen expressed by all tissue macrophages), CD11b (lymphocyte function-associated antigen), and CD14 (LPS receptor) [15,16]. Since hyalocytes express CD45, CD11b, and CD64 and do not react with CD68 antibodies (macrophages/microglia phenotype), we used CD64, GFAP (muller cells), and CRALBP/CK8 (retinal cells) as reliable markers for checking culture purity [15,31].

Second, the comparison of 3 d- and 6 d cultures highlighted that a specific protein signature is retained only in 3 d cultures. In fact, the biochemical analysis of conditioned media from 3 d cultures showed a protein signature similar to those of the untouched vitreous, except for the collagen composition that was significantly reduced by both cell separation and culturing [15]. By contrast, 6 d cultures showed a reorganization of the matrix (collagen, vimentin, hyaluronic acid) protein profile in their conditioned media probably due to an interplay of media components and hyalocyte stimulation to produce new mediators in a changed environment [15]. Since the contribution of hyalocytes inside the vitreous chamber is clear (homeostasis, parainflammation, inflammation/inflammaging), as hyalocytes mainly synthesize ECM (hyaluronan, glycoproteins, and collagen), regulate vitreous immunology, and modulate local inflammation, we verified if the culturing of hyalocytes might affect matrix composition. The results of this comparison showed that the characteristics of culturing did not massively influence the expression of these factors in 3 d cultures as compared to untouched ones, while 6 d-cultures showed a significant reduction in some mediators (collagen, VEGF, TGFβ1, bFGF) [3,32]. The biochemical profile was consistent with the molecular one. By secreting all isoforms of TGFβ and VEGF-A, hyalocytes can change and/or enrich vitreous collagen composition, enhance local contractile forces, causing tangential vitreo-retinal traction in the case of ERMs, and sustain vascular hyperpermeability (angiogenesis) [3,32]. Previous studies emphasized the dual role of hyalocytes in the protection and degeneration of retinal interphase upon different states (iatrogenic forms, autoimmune (diabetic) diseases, vascular, and aging (age-related macular degeneration)) [4,5,6]. Particularly, hyalocytes were associated with innate immune activation of complement fragments, promoting phagocytic activities of mononuclear cells and the oxidative stress-mediated response, providing additional explanations for their implication in the retraction of vitreoretinal interphase during diabetic retinopathies [2,3,6]. Herein, the expression of NGF and trkA^NGFR^ could support the homeostatic and neuroprotective roles of hyalocytes [33].

Third, the fact that hyalocytes can display some EMT properties, synthesize αSMA protein, and contract a collagen matrix is not new [28,34]. Previous studies reported the colocalization between CD163 and αSMA, suggesting that hyalocytes can transdifferentiate into myofibroblast-like cells [2]. Herein, the molecular analysis of cultured hyalocytes showed that the upregulation of αSMA transcripts was higher at 3 d than 6 d, supporting the data from the 3D gel contraction model. In fact, another novelty of our study is that collagen-embedded hyalocytes showed contractile properties in minimal as well as supplemented gels. The choice of NGF as a contractile-promoting factor was supported by the observation of trkA^NGFR^ on 3 d cultured hyalocytes. Our data on αSMA expression are in line with previous studies, highlighting the ability of hyalocytes to display a contractile phenotype in vitro and in vivo [3,15,35]. By itself, the EMT feature can be directly or indirectly mediated by the activation of Müller Cells, Retinal Pigment Epithelial (RPE), and the immune cells recruited from the vascular system (choroid vessel) and their ability to synthesize contractile mediators [36,37].

The limits of this study are related to the absence of flow-cytometer data to determine the percentage of hyalocytes and their early/late apoptotic state at 6 d. Unfortunately, these flow-based instruments are useful for high-density cell samples and do not appear suitable for a few cells such as the vitreous ones [38]. Indeed, the risk to retain the initial pathological properties is mandatory for these approaches and it appears more evident that all the efforts are granted in 3 d cultures than 6 d ones. Unfortunately, the presence of conditioned media characterized by the presence of FCS and some growth factors provides a good microenvironment to reverse a pathological phenotype [39]. Finally, being either local or systemic-driven, the recruitment and activation of hyalocytes imply the concomitant accumulation of inflammatory/profibrogenic/neurogenic mediators in the vitreous chamber that, in turn, would prompt the development and/or exacerbation of hyalocytes’ activities (matrix metabolism and tissue contraction), with the consequent distortion of the vitreous and retinal layers [2,3,25]. As known, hyalocytes belong to the monocyte/macrophage lineage and a plethora of cells populate the vitreous under pathological conditions with the risk of an improper cell selection. The high density of CD45/CD11a in vitreous cells coupled with αSMA expression significantly correlated with unfavorable prognostic factors and poor clinical outcomes in patients with vitreoretinal diseases [15].

## 5. Conclusions

It is clear that vitreous cells, and particularly hyalocytes, can typify the inflammatory state and contractile abilities at the vitreoretinal interface, representing, in the near future, a good tool for disease/therapy profiling. Certainly, the possibility of developing 3 d cultures might represent the first concept for advanced point-of-care lab-on-chip tests (precision medicine) [40,41]. Since the preservation of pathological properties might be of great interest for testing some functions in vitro, good and pure cellularity represents one of the cardinal points for developing in vitro assays. Under the physiological process, the removal of debris/death cells might be essential for isolating and culturing cells [42]. Under pathological events, EMT and apoptosis might occur alongside a heterogenic cell phenotype due to disease evolution, which allows a difficult cell separation [43]. Since primary hyalocytes might be a precious resource in the field of translational medicine, the finding of the present study would provide additional efforts in this direction and more experiments that are necessary to better characterize hyalocytes collected from pathological vitreous belonging to Macular Pucker and sorted according to specific clusters of differentiation. Morphological, biochemical, and transcriptomic profiles of these in vitro growing hyalocytes are underway to provide more information on these peculiar and interesting cells.

## Data Availability

Data are contained within the article.

## Supplementary Materials

The following supporting information can be downloaded at https://www.mdpi.com/article/10.3390/cells13221837/s1. Table S1: Organoleptic characteristics of the vitreous of the vitreous samples; Table S2: Antibodies (Abs) and primers (Gene).
