# Supplementary material for: Short-Term Culture of Human Hyalocytes Retains Their Initial Phenotype and Displays Their Contraction Abilities
Source: Cells. 2024 Nov 6;13(22):1837. doi: 10.3390/cells13221837 (PMC11592754; doi:10.3390/cells13221837)
Supplement: Supplementary file 1 [file cells-13-01837-s001.zip › cells-3264021-supplementary.pdf]

cells MDPI

Table S1. Organoleptic characteristics of the vitreous of the vitreal samples.

Code	PT	age	sex	VH	Cell density	plating	Total RNA (ng/µL)	Total protein (µg/µL)
**1**	136 V0	76	F	jelly	(++)	0d	155,32	3,20
**2**	156 V0	64	F	jelly	(+++)	0d	124,84	0,83
**3**	606 V0	66	M	jelly	(+++)	0d	179,92	0,85
**4**	126 V0	83	F	jelly	(++)	0d	21,53	3,2
**5**	226 V0	76	M	jelly	(+++)	0d	86,42	4,05
**6**	216 V0			jelly	(+++)	0d	10,87	3,60
**7**	235 V0	78	M	semi-jelly	(--)	0d	561,06	3,37
**8**	313 V0			semi-jelly	(++)	0d	170,74	
**9**	2361 V0	63	M	semi-jelly	(++)	0d	118,43	3,69
**10**	2362 V0	55	M	semi-jelly	(-/+)	0d	10,64	3,65
**11**	4071 V0	64	F	semi-jelly	(--)	0d	418,68	3,40
**12**	1008 V0	78	F	semi-jelly	(++)	0d	88,73	7,33
**13**	31071 V0	64	M	liquid	(--)	0d	109,05	
**14**	31072 V0	68	M	liquid	(-/+)	0d	123,55	7,35
**15**	6071 V0	71	M	liquid	(++)	0d	150,47	
**16**	6072 V0	87	F	liquid	(--)	0d	124,84	
**17**	20081 V0	71	M	liquid	(--)	0d	204,05	7,43
**18**	20082 V0	70	F	liquid	(--)	0d	160,60	7,43
**19**	132 V0	72	M	semi-jelly	(++)	0d	87,27	
**20**	7071 V1	72	F	jelly	(+++)	0d	14,31	3,44
**21**	3061 V1	75	F	jelly	(-/+)	0d	29,63	3,91
**22**	3062 V1	67	F	semi-jelly	(++)	0d	598,58	3,78

Legend: Retinal detachment Legend cells: (--), no detectable; (-/+), 0-10 cells / optic field; (+), 10-20 cells / optic field; (++), 20-30 cells / optic field; (+++), >30 cells / optic field; *, 2000cuts.

Table S2. Antibodies (Abs) and Primers (Gene).

A				
antibody	*code*	*specific*	*company*	*kDa*
huCD45	***sc -1178***	***hyalocytes***	***Santa Cruz Biotechnology***	***≈190kDa***
huCD11a	***11-210-C100***	***leukocytes***	***Exbio***	***≈41kDa***
huCD11b	***11-681-C100***	***Microglia and hyalocytes***	***Exbio***	***≈150kDa***
huCD74	***11-500-C100***	***macrophages***	***Exbio***	***≈30kDa***
huCD64	***11-644-C100***	***hyalocytes***	***Exbio***	***≈70kDa***
huCD14	***11-212-C100***	***macrophages***	***Exbio***	***≈65kDa***
huGFAP	***PA5-16-291***	***Glial cells***	***Invitrogen***	***≈50kDa***
huVimentin	***AB-82376***	***Fibroblast-like cells***	***Immunological Science***	***≈54kDa***
huαSMA	***A2547***	***Fibroblast-like cells***	***Sigma Aldrich***	***≈42kDa***

B			
Gene[a]	*References*	*Forward (5′-3′)*	*Reverse (3′-5′)*
CD45	*See [15]*	CTGACATCATCACCTAGCAG	TGCTGTAGTCAATCCAGTGG
CD11a	*See [15]*	TGAGAGCAGGCTATTTGGGTTAC	CGGCCCATGTGCTGGTAT
CD11b	*See [15]*	GGCCATGCACAGATACCAGGT	CTGGGGGTGCGATTTTCT
CD64	*See [15]*	GCCACAGAGGATGGAAATGT	CATGAAACCAGACAGGAGTGG
CD68	*See [15]*	TGGATTCATGCAGGACCTCC	CGCCATGTAGCTCAGGTAGACA

GFAP	*See [6]*	ACATCGAGATCGCCACCTAC	ACATCACATCCTTGTGCTCC
αSMA	*See [35]*	GAAGGAGATCACGGCCCTA	ACATCTGCTGGAAGGTGGAC
H3	*See [35]*	GCTGAGCTATGAGCCAAACC	TCGCCATTTCCATTAAGGTC
		GCTTCGAGAGATTCGTCGTT	GAAACCTCAGGTCGGTTTTG

[a] human sequences.
